# The impacts of nonnegative doctor portrayals on public evaluations and professional attractiveness in medicine

**DOI:** 10.1038/s41598-025-18847-5

**Published:** 2025-09-26

**Authors:** Qiwei Li, Zhefei Mao, Yuhui Shao, Yitong Huang, Qi Zhang, Jie Zhou

**Affiliations:** 1 Key Laboratory of Behavioral Science, Institute of Psychology, Chinese Academy of Sciences, Beijing, China; 2 Department of Psychology, University of Chinese Academy of Sciences, Beijing, China; 3 Department of Experimental Clinical and Health Psychology, Ghent University, Henri Dunantlaan 2, Ghent, 9000 Belgium; 4 Teachers College of Beijing Union University, Beijing, 100101 China; 5 Learning and Psychological Development Institution for Children and Adolescents, Beijing Union University, Beijing, 100101 China

**Keywords:** Doctors’ media portrayals, Doctor‒patient relationships, Stereotype content model, BIAS map, Psychology, Health occupations

## Abstract

This study explores the impacts of four common nonnegative media portrayals of doctors (i.e., science experts, angels in white, white-coated warriors, and vulnerable groups) on public evaluations (i.e.,stereotype content, emotional responses, and trust) and professional attractiveness (i.e.,willingness to marry or encourage child to become a doctor). Study 1 (*N* = 216) featured a between-participants design, revealing that the warrior and angel portrayals both led to more favourable ratings for warmth, competence, morality, admiration, reduced contempt, and trust than were observed in the control group. The warrior portrayal consistently received the highest scores in most dimensions, including professional attractiveness. The expert portrayal notably enhanced competence evaluations and reduced contempt, whereas the vulnerable portrayal elicited higher levels of sympathy but was associated with the lowest scores in most other dimensions. Study 2 (*N* = 320) featured a 3 (portrayal type; within-participants) × 6 (sequence; between-participants) mixed design, revealing that presentation order moderated the effects of such portrayals. The most effective sequence, angel–expert–vulnerable, elicited the highest overall evaluations, whereas the angel–vulnerable–expert sequence was least effective. These findings suggest that not only content but also sequence of portrayals can shape public attitudes towards doctors, thus highlighting relevant implications for health communication and media strategies.

**Supplementary Information:**

The online version contains supplementary material available at 10.1038/s41598-025-18847-5.

## Introduction

High-status professional groups such as physicians are not always perceived in entirely positive manners by the public. Although such groups are typically associated with competence, they are often viewed as lacking warmth^1^, which can strain doctor‒patient relationships. In countries such as China and India, such tensions have led to frequent conflicts^2–4^. Negative impressions and frequent conflicts may also reduce the attractiveness of the medical profession, thereby contributing to the global “doctor shortage”^5^. Therefore, building public trust and restoring the profession’s attractiveness are critical goals.

Exploring the ways in which the media shapes the portrayals of doctors could reveal viable ways of improving both doctor‒patient trust and the attractiveness of the medical profession. On the basis of an analysis of related reports, Wei and Chen^6^ identified three types of positive media portrayals of doctors: (1) The “experts” portrayal is presented in scientific popularization reports. In these reports, doctors are depicted as authoritative and knowledgeable professionals who translate complex medical knowledge into accessible language for the general public. For example, physicians are often featured in columns included in these reports to explain disease prevention strategies or debunk health myths. (2) The “angels in white” portrayal is presented in feature stories and recognition-oriented reports, and it focuses on dedication and healing. For example, profiles of physicians who work long hours in underserved regions or provide free care to disadvantaged patients exemplify this portrayal. (3) The “white-coated warriors” portrayal is a situational subtype of the “angels in white” portrayal that emerges most notably in times of crisis, such as in response to natural disasters or epidemics. In these narratives, doctors are portrayed as heroic figures who risk their own safety to treat patients under dangerous and high-pressure conditions. A fourth type of media portrayal is the “vulnerable doctor”, which presents doctors as individuals who face significant levels of physical, emotional, and systemic strain^7^. Unlike heroic or authoritative portrayals of doctors, this portrayal highlights challenges such as overwork, inadequate pay, and exposure to violence, thus reflecting ongoing structural issues in healthcare systems such as that of China. For instance, studies have reported that workplace violence affects between 42.2% and 83.3% of Chinese healthcare workers^8^, alongside long hours, limited pay relative to training, and increasingly tense doctor–patient relationships^9^. In urban hospitals, doctors may be required to see dozens of patients in a single morning, leaving only a few minutes per consultation. This high-pressure environment restricts meaningful communication and contributes to public frustration—often misattributed to physicians’ attitudes rather than systemic limitations. In recent years, to ease strained medical relationships, portrayals of vulnerable doctors have become increasingly common. This portrayal shifts the focus from doctors’ power to their precarious situation, thereby potentially eliciting empathy from the public^7^.

However, critical questions remain unanswered. Might this “vulnerable” portrayal decrease people’s faith in doctors by undermining perceptions of doctors’ ability? Could the frequent portrayal of doctors as “warriors” during pandemics prevent the public from encouraging family members to enter the medical profession? This study examines how these four types of nonnegative media portrayals of doctors influence the public’s social evaluations (in terms of stereotypes, emotions, and trust) and the perceived attractiveness of the medical profession.

## Literature review and problem statement

### The impacts of media portrayals of doctors on the public’s social evaluations

The term “media portrayal” refers to the portrayals that are constructed by mass communication media^10^. Media portrayals significantly shape public perceptions of doctors, who are members of a high-status profession that is closely linked to issues of trust, compliance, and career attractiveness. According to the stereotype content model (SCM), social groups are evaluated primarily in terms of warmth and competence^1^although morality was subsequently added as a key predictor of trust and cooperation^11^. The BIAS Map^12^ extends these evaluations to encompass emotional responses, including admiration, contempt, envy, and pity, which in turn influence individuals’ behavioural tendencies.

Wei and Chen^6^ identified three types of nonnegative doctor portrayals in Chinese media: (1) “experts,” who convey authority and competence in science reporting; (2) “angels in white,” who exhibit compassion and dedication in the context of providing daily care; and (3) “white-coated warriors,” a crisis-specific version of the angel portrayal that highlights doctors’ bravery and sacrifice in emergency situations. These portrayals are expected to strengthen perceptions of doctors’ competence, morality, and warmth; increase admiration; reduce contempt; and ultimately strengthen public trust^3^.

In contrast, the “vulnerable doctor” portrayal emphasizes doctors’ exposure to violence, overwork, and inadequate compensation—issues that are increasingly common in media throughout China, India, and Iraq^7,13–15^. While such portrayals may elicit sympathy by humanizing doctors, repeated emphasis on the low power and status of this group may unintentionally undermine perceptions of doctors’ competence^1,16^. Sympathy is typically directed at groups that are viewed as warm but less competence, which may foster benevolence without respect^12^. Furthermore, trust in physicians depends not only on their perceived moral integrity but also on people’s confidence in their ability to manage risk and uncertainty^3,16^. Overexposure to doctors’ helplessness may signal reduced control or efficacy, thereby ultimately weakening public trust.

Accordingly, the following hypotheses are proposed:

H1a: Media portrayals of doctors as “experts,” “angels in white,” and “white-coated warriors” increase the public’s ratings of doctors’ warmth, competence, and morality; enhance admiration; reduce contempt; and increase trust in doctors.

H1b: The “vulnerable” portrayal of doctors reduces perceived competence, increases sympathy, and decreases trust in doctors.

### The impacts of portrayals of doctors on the attractiveness of the medical profession

The “doctor shortage”—which is currently a notable predicament in the medical field—is likely to lead to a decline in the attractiveness of the medical profession. For many years, surveys conducted by the Chinese Medical Doctor Association have included a question regarding whether doctors would “like their children to become doctors,” which is believed to provide a comprehensive evaluation of the professional environment and societal esteem of doctors^17^, thus indicating the attractiveness of this profession. Accordingly, given that the primary individuals on which this study focuses are employed adults, their intentions regarding their partners’ and children’s professional choices might represent their opinions concerning a profession more accurately. Therefore, we use the public’s preference for a medical spouse and their desire for their children to pursue medicine as indicators of the attractiveness of the medical profession in an effort to discover solutions to the “doctor shortage.” Especially in China, students’ career aspirations are largely influenced by their families. Consequently, an increase in the public’s preference for medical spouses and their desire for their children to enter the field of medicine might increase the attractiveness of the medical profession, thereby potentially increasing the number of medical practitioners, reducing existing doctor turnover rates, and ameliorating the “doctor shortage.”

However, the question whether the portrayals of “white-coated warriors” and “vulnerable group” enhance the public’s preference for medical spouses and their desire for their children to enter the field of medicine in comparison to the portrayals of “experts” and “angels in white” remains unanswered. The portrayal of doctors as “warriors” may reflect professional risk while simultaneously highlighting the principle of sacrifice. Especially during pandemics, such an portrayal could strengthens perceptions of the risks associated with this profession, thus leading the public to avoid the medical profession in their choices of spouses or careers for their children. However, the “warriors” portrayal can also significantly enhance the professional reputation and status of medical workers, thereby potentially increasing the attractiveness of the medical profession. Surveys have reported that after the outbreak of COVID-19, parents of medical students significantly increased their desire for their children to become doctors^18^. The “vulnerable group” portrayal, on the other hand, could decrease the perceived social status of doctors and reduce the attractiveness of the profession. Accordingly, we propose the following hypotheses:

H2a: The media portrayals of doctors as “experts,” “angels in white,” and “white-coated warriors” can increase the public’s preference for medical spouses and their willingness for their children to become doctors.

H2b: The media portrayal of doctors as a “vulnerable group” reduces the public’s preference for medical spouses and their willingness for their children to become doctors.

Moreover, the question of which type of nonnegative media portrayal has the strongest overall impact when multiple outcome dimensions—namely, stereotype content, emotional responses, trust and professional attractiveness—are considered simultaneously remains unanswered. Given that these dimensions are interrelated yet distinct^12,19^, understanding the relative efficacy of each such portrayal is critical with respect to the development of targeted media strategies. We thus propose our first research question as follows:

RQ1: Which type of nonnegative doctor portrayal has the most favourable overall effect across the cognitive, emotional, trust-related and professional dimensions?

Furthermore, real-world media environments rarely present a single, consistent portrayal of doctors. In daily life, individuals are frequently exposed to multiple and sometimes conflicting portrayals, such as alternating reports of doctors as heroic frontliners and overworked victims. Previous research on order effects and message framing in this context has suggested that the sequence and combination of exposure can meaningfully shape audience perceptions^20,21^. However, little is known regarding how combinations of different doctor portrayals and the order in which they are presented influence public evaluation.

For example, encountering the “angel in white” portrayal before the “expert” portrayal may activate warmth-based admiration and emotional connection, thereby enhancing the perceived credibility and influence of the subsequent portrayal. Conversely, viewing a “vulnerable doctor” portrayal before the “expert” portrayal may evoke sympathy but also reduce perceived competence, which could decrease the persuasiveness of the expert portrayal and reduce downstream trust. These possibilities highlight the importance of examining interaction effects between portrayal type and presentation sequence at both the theoretical and practical levels.

RQ2: Do different combinations and presentation sequences of nonnegative doctor portrayals have differential effects on public perceptions of doctors?

Addressing these questions can not only improve our theoretical understanding of media-based impression formation and stereotype updating but also offer practical implications that can be used to improve doctor–patient relationships and the public portrayal of the medical profession.

## Study 1

Study 1 was conducted from November 5 to 15, 2020 after receiving approval from the Ethics Subcommittee of Institute of Psychology, Chinese Academy of Sciences (approval no. H19050, issued 26 October 2019). The study was conducted in accordance with the principles of the Declaration of Helsinki, all participants provided written informed consent before any study procedures began. They received a concise description of the study’s aims, were assured that their responses would remain anonymous and would be used exclusively for scientific purposes, and were explicitly reminded that they could withdraw at any time without penalty.

### Presurvey

By reference to the definitions of four types of common nonnegative doctor portrayals and the summary of relevant typical reports produced by Wei and Chen^6^ and An^9^we compiled the materials that we used to manipulate doctor media portrayals and conducted a preexperiment. The contents of the “experts” portrayal focused on the deeds of famous domestic science popularization platforms and relevant experts. The contents concerning the “angels in white” portrayal pertained to the advanced deeds of doctors who won the “China Physician’s Day” award. The contents associated with the “white-coated warriors” portrayal covered various touching deeds on the part of medical personnel on the frontlines of epidemic prevention. The contents related to the “vulnerable” portrayal included a survey report that described the current situation faced by doctors, which involves high levels of work intensity, low incomes, and tense doctor–patient relationships. The material associated with each portrayal contained an introduction to the overall situation of the corresponding content and a description of typical cases; this approach is in line with actual news reports, which tend to focus on typical characters in an effort to reflect group phenomena and combine individual portrayals with group portrayals. The materials are available in Appendix 1. For privacy protection purposes, the images used in the portrayals of the expert, angel, and vulnerable doctor conditions have been replaced with researcher-designed posters from Study 2 that contain no identifiable facial features. The accompanying text content remained consistent across both studies.

A convenience sampling approach was used to recruit a total of 64 individuals (18 males) via the WeChat group used in the presurvey; the average age of participants was 25 years (*SD* = 8.92). These participants were asked to read 4 news items, which were presented in random order, and to select the doctor portrayal shaped by the news. Moreover, the participants were required to write down the surname of the doctor who was described in detail in the news as a seriousness check. Ultimately, 55 questionnaires were retained. Statistical information revealed that the successful shaping ratio for the “expert” and “warrior” portrayals was 100%, the corresponding ratio for the “vulnerable” portrayal was 94.5%, and the ratio for the “angel” portrayal was 92.7%. Thus, the manipulation of the four types of doctor media portrayals via these experimental materials was successful.

### Formal survey

The formal study featured a single-factor, between-participants design, and a control group was included in addition to the 4 experimental groups. In the control group, participants read a short, emotionally neutral news-style story that described an ordinary person who had lost their backpack on a Tuesday afternoon. The passage concluded with a practical reminder that encouraged readers to take care of their personal belongings in public places. This material was entirely unrelated to doctors or healthcare and was carefully matched with the experimental texts in terms of length and formatting.

The required sample size was estimated with the assistance of G*Power 3.1 software^22^; the main effect was set to achieve 80% test power, and the effect size was set to 0.25. The results revealed that the total sample size needed was 200. Ultimately, a convenience sampling approach was used to collect 216 questionnaires via Questionnaire Star (www.wjx.cn, an online survey platform in China); a total of 39 participants were included in the control group, 42 in the expert group, 47 in the angel group, 42 in the warrior group, and 46 in the vulnerable group. The average age of the participants was 34.67 years (*SD* = 7.05), and the sample included 88 males. First, the corresponding materials were presented to each group of participants. They were asked to describe the character portrayed in the news in two words as a manipulation check. Subsequently, the participants’ stereotypes of doctors and other research variables were measured. Finally, various demographic variables, including gender, age, education, annual income, and physical health status, were measured as control variables.

### Measurement tools

All scales used in this study were scored on a 5-point Likert scale, in which context 1 indicated complete disagreement and 5 indicated complete agreement (See Appendix 2).

#### Stereotype content scale

The stereotype content model questionnaire developed by Guan and Cheng^23^ was used in this research. The instructions provided were as follows: “In your opinion, members of the doctor group are…” This scale included a total of 6 questions. The Cronbach’s α coefficient for this scale was 0.75.

#### Intergroup emotions

Four items drawn from Guan and Cheng^23^ were used to measure the intensity of the contempt, admiration, sympathy, and envy exhibited by the participants towards the doctor group with the aim of exploring the roles played by these four emotions separately. The question asked with regard to this instrument was as follows: “When you think about the doctor group, how strongly do you experience the following emotional reactions?” The participants responded to this question on a scale ranging from 1 (“not at all”) to 5 (“very strongly”).

#### Trust in doctors

This research relied on Deng’s^24^ single-dimensional patient trust scale, although some items were deleted, and two items that were newly developed for this research were added, i.e., “Overall, I trust doctors” and “I can entrust my life to doctors without hesitation”; the scale included a total of 6 items. The Cronbach’s α coefficient for this scale in this study was 0.85.

#### Willingness to marry a doctor

A single item was used to measure this factor: “Are you willing to marry a doctor?”

#### Willingness to allow children to become doctors

A single item was used to measure this factor: “Would you like your child to study medicine in the future?”

### Results of the survey

All analyses were conducted using SPSS 22.0. We conducted two-tailed Pearson correlation analyses to examine the relationships among key variables (see Appendix 3 Table S1). The correlation coefficient between the warmth and morality dimensions was only 0.58, thus verifying the hypothesis concerning the separation of the warmth and morality dimensions. Notably, correlation analyses revealed that level of education was positively associated with admiration for doctors (*r* = .22, *p* = .001). In addition, self-reported physical health was positively correlated with trust (*r* = .18, *p* = .010).

To examine potential group-level differences among the conditions, a one-way analysis of variance (ANOVA) was conducted for each demographic variable. The results indicated that among all the variables measured in this context, only physical health status exhibited significant differences across the five groups, F(4, 211) = 2.56, *p* = .040. No significant differences were observed in age, gender, education, or income. Therefore, physical health status was included as a covariate in subsequent one-way ANOVAs to control for its potential influence on the outcome variables.

One-way ANOVAs controlling for physical health status were performed to test group differences (see Table 1). In terms of stereotypes regarding doctors, evaluations of doctors’ warmth and morality were highest in the warrior group and lowest in the vulnerable group. Significant differences were observed among the five groups in terms of their evaluations of the morality of doctors, *p* = .003, and marginally significant differences were observed in terms of competence evaluations, *p* = .076. Least significant difference (LSD) post hoc comparisons revealed that participants in the warrior and angel groups rated doctor morality significantly higher than did those in the vulnerable group (*p* ≤ .012). In terms of competence, participants in all three nonnegative portrayal groups (i.e., expert, angel, and warrior) rated doctors as significantly more competent than did participants in the control group (*p*s ≤ 0.019), whereas participants in the vulnerable group did not differ significantly from those in the control group.

**Table 1:** Descriptive statistics and results of the ANOVA conducted for study 1 (M(SD)).

Dependent variables		Control	Expert	Angel	Warrior	Vulnerable	F	Partial η^2^
Public’s social evaluations	Warmth	3.92 (0.11)	3.98 (0.11)	3.94 (0.10)	**4.12 (0.11)**	**3.73 (0.10)**	1.79	0.03
	Competence	**4.07 (0.10)**	4.39 (0.09)	**4.40 (0.09)**	4.39 (0.09)	4.29 (0.09)	2.15†	0.04
	Morality	4.03 (0.09)	4.16 (0.09)	4.30 (0.08)	**4.43 (0.09)**	**4.00 (0.09)**	4.24**	0.08
	Contempt	**1.48 (0.09)**	1.16 (0.09)	1.17 (0.08)	**1.11 (0.09)**	1.35 (0.08)	3.04*	0.06
	Admiration	**4.08 (0.14)**	4.21 (0.13)	4.45 (0.12)	**4.65 (0.13)**	4.28 (0.12)	2.87*	0.05
	Sympathy	2.79 (0.17)	**2.76 (0.16)**	2.98 (0.15)	2.95 (0.16)	**3.30 (0.15)**	1.89	0.04
	Envy	**1.38 (0.13)**	**1.73 (0.13)**	1.43 (0.12)	1.45 (0.13)	1.47 (0.12)	1.18	0.02
	Trust	3.73 (0.11)	3.78 (0.10)	3.88 (0.10)	**3.92 (0.10)**	**3.69 (0.10)**	0.93	0.02
Attractiveness of the medical profession	Married	3.67 (0.14)	3.52 (0.14)	3.60 (0.13)	**3.71 (0.14)**	**3.31 (0.13)**	1.45	0.03
	Children	3.82 (0.16)	3.86 (0.15)	**3.56 (0.15)**	**3.94 (0.15)**	3.69 (0.15)	1.02	0.02

In each row, bolded values denote the lowest and highest scores for that variable across all conditions.

†*p*<.10,*p<.05,**p<.01,***p<.001 (these abbreviations are also used hereinafter).

In terms of participants’ emotions towards doctors, significant group differences were observed in both contempt (*p* = .018) and admiration (*p* = .024). Post hoc comparisons revealed that contempt was significantly lower among participants in the expert, angel, and warrior groups than among those in the control group (*p* ≤ .012), whereas the vulnerable group exhibited no significant differences in this regard. Admiration was significantly higher among participants in the warrior and angel groups than among those in the control group (*p* ≤ .043). No significant differences were observed in sympathy or envy among the five groups (*ps* > 0.05). However, descriptively, sympathy was highest in the vulnerable group and lowest in the expert group, whereas envy was highest in the expert group and lowest in the control group.

The differences among the five groups in trust in doctors, willingness to marry a doctor, and willingness to allow children to become doctors were not significant. However, the trust in doctors and willingness to marry a doctor reported by participants who read the news associated with the vulnerable portrayal were the lowest among the five groups, and the willingness to allow children to become doctors was also lower than that of participants in the control group. Moreover, trust in doctors, willingness to marry a doctor, and willingness to allow children to become doctors were the highest in the warrior group among all five groups.

### Discussion

Study 1 revealed that nonnegative media portrayals of doctors have distinct effects on public perceptions. Both the warrior and the angel in white portrayals significantly enhanced evaluations of doctors’ warmth, competence, morality, admiration, and trust while reducing contempt in comparison with the control group. The warrior portrayal consistently received the highest scores, including in terms of professional attractiveness, thus suggesting its broad and strong positive impact.

The expert portrayal mainly improved competence and reduced contempt, but it had weaker emotional and relational effects. These findings are in line with research that has reported that competence alone may not elicit admiration unless it is paired with warmth or morality^12^. In contrast, the vulnerable portrayal increased sympathy but was associated with lower scores on most other dimensions, thus indicating the potential risk of overemphasizing hardship in media narratives.

One limitation of Study 1 is the use of a convenience sample, which may affect the generalizability of the findings. The sample was relatively small and skewed toward younger and female participants, reflecting the demographic profile of online survey respondents in China. Moreover, the single-factor, between-participants design used in this study offers a degree of internal validity by isolating the effects of each portrayal. However, the question of whether these effects persist when individuals are exposed to multiple portrayals, as is common in real-world media environments, remains unanswered. A within-participants design, in which the same participants are presented with different framings, could simulate natural exposure conditions more accurately and provide a more nuanced understanding of comparative and sequential effects in this context.

## Study 2

Study 2 aimed to investigate how different combinations and presentation sequences of doctor portrayals influence public perceptions. This study was conducted in March 2025, shortly after the COVID-19 pandemic. To avoid potential confounding effects pertaining to pandemic-related emotions and memories, the “white-coated warrior” portrayal was excluded from the design.

To approximate real-world exposure more closely and address the limitations of the between-participants design used in Study 1, Study 2 employed a 3 (doctor portrayal: expert vs. angel vs. vulnerable; within-participants) × 6 (presentation order; between-participants) mixed factorial design. The participants were randomly assigned to one of six reading sequences and presented with all three portrayals of doctors; however, these portrayals were shown in different orders depending on group assignment. After the participants read each passage, they completed the same set of measures used in Study 1 to assess stereotypes, emotions, trust, and professional attractiveness.

A priori power analysis conducted with the assistance of G*Power (effect size = 0.10, power = 0.80) indicated a minimum sample size of 276. In total, 320 valid responses were collected via Credamo (https://www.credamo.com/), an online survey platform. The participants’ mean age was 36.16 years (SD = 13.62); 143 participants (44.1%) were male, and 41.2% were between the ages of 40 and 69 years, thus enhancing the generalizability of the findings of this research.

### Results

We first conducted correlation analyses using SPSS 22.0 among the outcome variables (see Appendix 3 Tables S2a–c). The correlation coefficient between warmth and morality was less than 0.67, thus supporting the theoretical distinction between these two dimensions of stereotype content. As in Study 1, level of education was positively associated with overall admiration for doctors (*r*s ≥ 0.09, *p*s ≤ 0.098), and self-reported physical health was positively correlated with trust (*r*s ≥ 0.16, *p*s ≤ 0.003). In addition, one-way ANOVAs revealed no significant group differences in gender, age, education, household income, or health status across the six reading order conditions (*p*s > 0.05), thus indicating successful randomization. Therefore, no control variables were included in the main analyses.

**Table 2:** Results of the ANOVA conducted for study 2.

			F	*p*	Partial η2
Public’s social evaluations	Warmth	Portrayal	55.429	< 0.001	0.150
		Sequence	2.165	0.058	0.033
		Portrayal*sequence	4.013	< 0.001	0.060
	Competence	Portrayal	29.411	< 0.001	0.086
		Sequence	0.830	0.529	0.013
		Portrayal*sequence	29.411	< 0.001	0.086
	Morality	Portrayal	30.377	< 0.001	0.088
		Sequence	1.352	0.242	0.021
		Portrayal*sequence	0.644	0.776	0.010
	Contempt	Portrayal	0.305	0.737	0.001
		Sequence	1.297	0.265	0.020
		Portrayal*sequence	2.577	0.005	0.039
	Admiration	Portrayal	15.116	< 0.001	0.046
		Sequence	1.527	0.181	0.024
		Portrayal*sequence	3.506	< 0.001	0.053
	Sympathy	Portrayal	113.589	< 0.001	0.266
		Sequence	2.078	0.068	0.032
		Portrayal*sequence	6.816	< 0.001	0.098
	Envy	Portrayal	1.251	0.286	0.004
		Sequence	2.350	0.041	0.036
		Portrayal*sequence	4.903	< 0.001	0.072
	Trust	Portrayal	24.810	< 0.001	0.073
		Sequence	1.316	0.257	0.021
		Portrayal*sequence	11.376	< 0.001	0.153
Attractiveness of the medical profession	Married	Portrayal	12.889	< 0.001	0.039
		Sequence	1.726	0.128	0.027
		Portrayal*sequence	1.081	0.375	0.017
	Children	Portrayal	24.846	< 0.001	0.073
		Sequence	3.540	0.004	0.053
		Portrayal*sequence	1.130	0.338	0.018

**Fig. 1:**
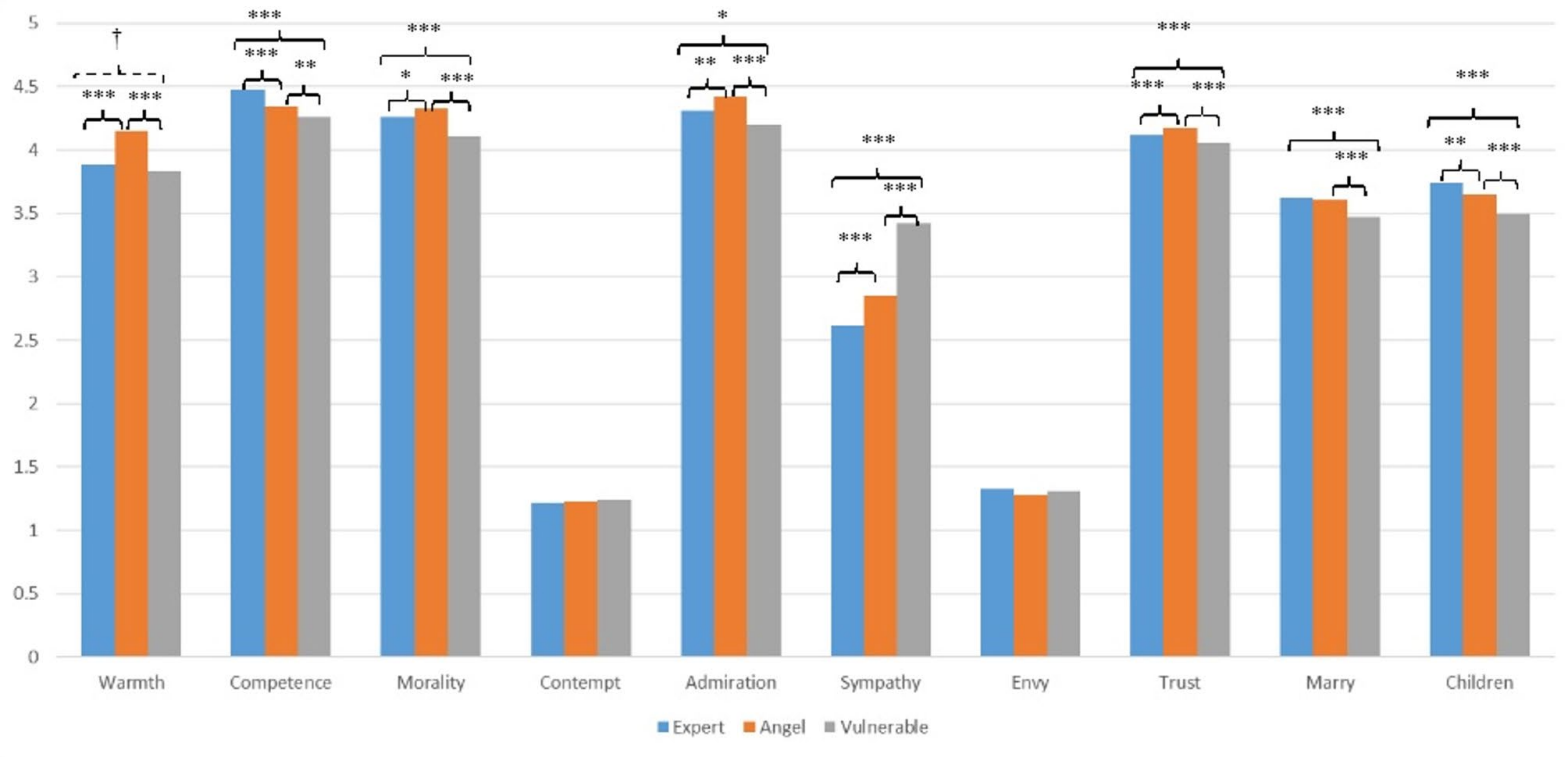
Mean scores of the three doctor portrayals on each outcome variable in study 2.

Mixed-design ANOVAs revealed significant within-participant effects of doctor portrayal on all outcome variables with the exceptions of contempt and envy (see Table 2), *F*s ≥ 12.889, *p*s < 0.001. As indicated in the post hoc comparisons (see Fig. 1), the participants rated doctors’ competence, their own willingness to marry a doctor, and their willingness to allow their children to become doctors the highest after they read the materials pertaining to the expert portrayal. In contrast, the angel portrayal elicited the highest ratings in terms of warmth, morality, admiration, and trust. The vulnerable portrayal elicited the highest level of sympathy towards doctors but was consistently associated with the lowest scores across all other outcomes with the exceptions of contempt and envy.

The interaction effect between doctor portrayal and reading order was significant for all variables with the exceptions of participants’ morality evaluations, willingness to marry a doctor and willingness to allow children to become doctors (see Table 2). A comparison of the outcome scores across the six presentation sequences revealed clear differences in effectiveness (see Table 3). The sequence angel–expert–vulnerable yielded the most favourable results, as this order was associated with the highest ratings with respect to 13 outcome variables. This sequence was followed by the vulnerable–angel–expert sequence, which received the highest ratings in terms of 7 variables. In contrast, the angel–vulnerable–expert sequence resulted in the lowest ratings in terms of 19 variables, thus identifying it as the least effective sequence. The vulnerable–expert–angel sequence ranked second to last, as it was associated with the lowest scores with respect to 3 variables.

**Table 3:** Descriptive statistics regarding all the conditions in study 2 (M(SD)).

		E-A-V	E-V-A	A-E-V	A-V-E	V-E-A	V-A-E
Warmth	Expert	3.94 (0.62)	3.81 (0.58)	**4.01 (0.56)**	**3.80 (0.81)**	3.89 (0.61)	3.85 (0.59)
	Angel	**4.31 (0.62)**	4.28 (0.64)	4.09 (0.64)	**3.81 (0.96)**	4.27 (0.60)	4.11 (0.63)
	Vulnerable	3.99 (0.53)	3.73 (0.66)	**4.04 (0.60)**	**3.65 (0.92)**	3.79 (0.60)	3.75 (0.64)
Competence	Expert	4.46 (0.47)	**4.41 (0.50)**	4.51 (0.48)	4.45 (0.64)	4.44 (0.60)	**4.59 (0.50)**
	Angel	4.39 (0.49)	**4.43 (0.44)**	4.28 (0.51)	**4.18 (0.69)**	4.31 (0.47)	4.42 (0.52)
	Vulnerable	4.27 (0.58)	4.27 (0.52)	4.21 (0.57)	**4.15 (0.67)**	**4.35 (0.49)**	4.29 (0.56)
Morality	Expert	**4.34 (0.59)**	4.23 (0.56)	4.31 (0.50)	**4.14 (0.69)**	4.21 (0.60)	4.33 (0.46)
	Angel	**4.41 (0.59)**	4.30 (0.55)	4.35 (0.57)	**4.16 (0.79)**	4.41 (0.52)	4.33 (0.47)
	Vulnerable	**4.24 (0.53)**	4.07 (0.54)	4.17 (0.58)	**3.97 (0.78)**	4.09 (0.56)	4.12 (0.45)
Contempt	Expert	**1.35 (0.67)**	**1.13 (0.40)**	1.15 (0.42)	1.23 (0.43)	1.26 (0.56)	1.18 (0.64)
	Angel	1.20 (0.49)	1.19 (0.49)	1.23 (0.61)	**1.44 (0.83)**	1.19 (0.44)	**1.16 (0.60)**
	Vulnerable	1.18 (0.43)	**1.15 (0.36)**	**1.15 (0.42)**	**1.38 (0.75)**	1.37 (0.68)	1.18 (0.48)
Admiration	Expert	4.22 (0.81)	4.21 (0.64)	**4.56 (0.61)**	**4.08 (0.97)**	4.33 (0.70)	4.45 (0.63)
	Angel	4.47 (0.81)	4.50 (0.67)	**4.52 (0.61)**	**4.15 (0.80)**	4.46 (0.69)	4.40 (0.71)
	Vulnerable	4.29 (0.79)	4.13 (0.79)	**4.38 (0.66)**	4.25 (0.86)	4.09 (0.71)	**4.05 (0.68)**
Sympathy	Expert	**2.38 (0.95)**	**2.38 (0.93)**	2.67 (1.22)	**2.87 (0.84)**	2.67 (0.97)	2.78 (0.96)
	Angel	**2.53 (1.07)**	2.94 (0.98)	2.90 (1.03)	2.92 (0.97)	2.72 (1.00)	**3.07 (1.07)**
	Vulnerable	3.47 (1.12)	3.50 (1.04)	**3.94 (0.85)**	3.56 (0.96)	**2.93 (1.10)**	3.15 (0.93)
Envy	Expert	1.25 (0.55)	1.40 (0.66)	1.33 (0.68)	1.40 (0.75)	**1.43 (0.72)**	**1.16 (0.42)**
	Angel	1.13 (0.39)	**1.12 (0.32)**	1.38 (0.66)	**1.48 (0.85)**	1.30 (0.54)	1.25 (0.55)
	Vulnerable	**1.11 (0.32)**	1.23 (0.51)	1.12 (0.32)	1.40 (0.63)	**1.52 (0.77)**	1.49 (0.74)
Trust	Expert	4.02 (0.58)	**3.94 (0.50)**	**4.30 (0.45)**	4.13 (0.58)	4.11 (0.58)	4.24 (0.42)
	Angel	4.17 (0.58)	4.18 (0.48)	4.24 (0.48)	**4.01 (0.61)**	**4.24 (0.53)**	4.22 (0.43)
	Vulnerable	4.09 (0.59)	4.04 (0.50)	**4.26 (0.45)**	4.01 (0.62)	4.01 (0.55)	**3.97 (0.42)**
Married	Expert	3.64 (0.97)	**3.44 (0.92)**	3.85 (1.07)	3.42 (1.07)	3.50 (0.91)	**3.85 (0.97)**
	Angel	3.64 (1.03)	3.52 (0.98)	3.71 (1.09)	**3.38 (1.16)**	3.63 (0.94)	**3.75 (1.02)**
	Vulnerable	3.47 (1.12)	3.29 (1.00)	**3.71 (1.19)**	**3.23 (1.08)**	3.37 (0.90)	**3.71 (1.05)**
Children	Expert	3.78 (0.99)	3.63 (0.95)	**4.17 (0.81)**	**3.29 (1.11)**	3.74 (0.92)	3.80 (1.01)
	Angel	3.65 (1.08)	3.50 (1.04)	**4.02 (0.87)**	**3.40 (1.16)**	3.63 (0.94)	3.69 (0.96)
	Vulnerable	3.60 (1.08)	3.29 (1.11)	**3.87 (0.99)**	**3.10 (1.29)**	3.57 (1.02)	3.55 (1.09)

The sequence label in the first row (e.g., E-A-V) indicates that participants read the three news items in the order Expert–Angel–Vulnerable; analogous abbreviations apply to the remaining conditions. In each row, bolded values denote the lowest and highest scores for that variable across all conditions.

### Discussion

Study 2 extended the findings of Study 1 by employing a within-participants design and introducing reading order as a between-participants factor. The results of this study reinforced the previous findings indicating that different portrayals of doctors elicit distinct responses. The expert portrayal enhanced perceptions of competence and professional attractiveness, whereas the angel portrayal most strongly increased ratings of warmth, morality, admiration, and trust. The vulnerable portrayal consistently elicited the highest levels of sympathy but the lowest scores with regard to most other outcomes.

In addition, significant interaction effects between doctor portrayal and reading order were identified for most variables, thus indicating that the sequence in which portrayals are presented can meaningfully influence public perceptions. While morality evaluations and participants’ willingness to marry a doctor were unaffected by order, other outcomes varied considerably. Notably, the angel–expert–vulnerable sequence was associated with the most favourable overall evaluations, whereas the angel–vulnerable–expert sequence was linked with the least favourable outcomes. These findings suggest that not only the type of media portrayals but also the order in which they are presented can shape public attitudes towards doctors.

## General discussion

This research explored how different nonnegative media portrayals of doctors, i.e., experts, angels in white, white-coated warriors, and vulnerable doctors, influence public perceptions across different types of stereotype content, emotional responses, trust, and professional attractiveness. The two studies conducted for this research revealed that both the type and order of portrayals shaped audience evaluations significantly.

In single-exposure contexts (Study 1), the “white-coated warrior” portrayal elicited the most broadly positive responses, including the highest ratings of warmth, morality, admiration, trust, and willingness to marry or support children’s decision to become doctors. However, as this portrayal is typically linked with public health crises, it may have limited relevance for everyday messaging. The “angel in white” portrayal, which is a more common representation, also significantly increased warmth-, morality-, and trust-related outcomes, thus suggesting that it may be used as a practical tool to strengthen day-to-day doctor–patient relations.

In Study 2, in which participants were exposed to all three portrayals in varying orders, the “expert” portrayal proved to be the most effective way of enhancing perceived competence and professional attractiveness, especially when it was presented after the angel portrayal. This pattern is in line with previous research that has reported that initial exposure to warmth-based or emotionally positive content can increase individuals’ receptivity to subsequent competence-based information by facilitating affective priming and reducing defensive processing^25^. The angel–expert–vulnerable sequence led to the highest scores in multiple dimensions. One explanation for this finding is that the angel portrayal first activates affective trust and moral resonance, which increases receptivity to the expert frame, thereby reinforcing both emotional and cognitive evaluations. When the vulnerable portrayal appears last, its emotional tone may add empathy without undermining previous impressions, which is consistent with dual-processing models that highlight the integration of affective and analytic cues^26^.

In contrast, the least effective sequence, i.e., angel–vulnerable–expert, was associated with the lowest ratings in terms of most outcomes. This pattern may be to the result of emotional fatigue from two consecutive emotionally heavy portrayals (i.e., angel and vulnerable), which could reduce engagement with the third, more rational expert frame. Moreover, the shift from selfless dedication (angel) to helplessness (vulnerable) may decrease doctors’ perceived status and competence, thus making the expert portrayal appear to be less credible or even dissonant. This result is consistent with the findings of previous research indicating that exposure to weakness or loss of status can contaminate subsequent impressions, thereby weakening evaluations of competence and credibility^27,28^. Such mismatches may weaken the overall impact of such a message, particularly when emotional saturation precedes analytical content.

Notably, although the vulnerable portrayal could effectively increase sympathy, in consistently underperformed in terms of other dimensions. While these effects were often nonsignificant, this pattern highlights the need for caution in this context. An excessive emphasis on doctors’ hardship may humanize them but also risk weakening perceptions of their competence and trustworthiness, thus echoing the findings of previous studies on paternalistic stereotypes^12^.

Moreover, this research supports the conceptual distinction between morality and warmth in the context of stereotype content, thus reinforcing the claim that morality is an independent and central dimension of social evaluation^11^. We also found that level of education was positively associated with admiration for doctors, thus suggesting that the medical profession remains attractive to more highly educated individuals, who are also more likely to represent potential partners or candidates for the profession. This finding may have positive implications regarding the ongoing physician shortage. In addition, physical health status was positively linked to trust in doctors, thus highlighting the role of involvement in the process of shaping trust. Individuals whose health is poorer may be more motivated to seek, evaluate, and trust medical information. This finding suggests that the effects of media portrayals may vary depending on audience involvement, which represents an important direction for future research.

## Limitations and future research

Several limitations of this research should be acknowledged. First, in real-world media environments, the framing strategies used in this study are rarely employed in isolation. Competing narratives often coexist and compete to attract public attention, which may significantly influence how audiences interpret and respond to media portrayals. As a result, the controlled exposure used in our experiments may not have fully captured the complexity of real-life information processing.

Second, the generalizability of the findings of this research across different cultural and institutional contexts is limited. Media portrayals and public perceptions of doctors are likely to be shaped by the structure of the local healthcare system, norms regarding the medical profession, and cultural attitudes towards authority and care. While the portrayals examined in this context are rooted in Chinese media discourse, the underlying categories (such as competence, morality, vulnerability, and heroism) may also apply to doctors working in other countries or even to members of other high-status professional groups. Future researchers should explore how these portrayals function in diverse national and professional contexts.

## Supplementary Information

Below is the link to the electronic supplementary material.

Supplementary Material 1

## Data Availability

Open data & materials: The data that support the findings of this study and the information needed to reproduce all of the methodology reported in this context are openly available via the Open Science Framework (OSF) at https://osf.io/743dj/?view_only=e9904e8952534a8eab1a719684f5a145.
