# Supplementary material for: The impacts of nonnegative doctor portrayals on public evaluations and professional attractiveness in medicine
Source: Sci Rep. 2025 Sep 26;15:33154. doi: 10.1038/s41598-025-18847-5 (PMC12475096; doi:10.1038/s41598-025-18847-5)
Supplement: Supplementary file 1 — Supplementary Material 1 [file 41598_2025_18847_MOESM1_ESM.docx]

**Contents**

[Appendix 1 Experimental Materials for Four Non-Negative Doctor Portrayals and the Control Condition 2](#_Toc195618656)

[[Expert Group] 2](#_Toc195618657)

[[Angel Group] 5](#_Toc195618659)

[[Warrior Group] 8](#_Toc195618660)

[[Vulnerable Group] 11](#_Toc195618661)

[[Control group] 14](#_Toc195618662)

[Appendix 2 Study 1 Questionnaire 16](#_Toc195618663)

[[Chinese version] 16](#_Toc195618664)

[[English version] 18](#_Toc195618665)

[Appendix 3: Pearson Correlation Tables for Study 1 and Study 2 20](#_Toc195618666)

[Table S1. Pearson Correlation Tables for Study 1 20](#_Toc195618667)

[Table S2a. Correlation Matrix – Expert Portrayal Variables for Study 2 21](#_Toc195618668)

[Table S2b. Correlation Matrix – Angel Portrayal Variables for Study 2 22](#_Toc195618669)

[Table S2c. Correlation Matrix – Vulnerable Portrayal Variables for Study 2 23](#_Toc195618670)

### Appendix 1 Experimental Materials for Four Non-Negative Doctor Portrayals and the Control Condition

**[Expert Group]**-Chinese version


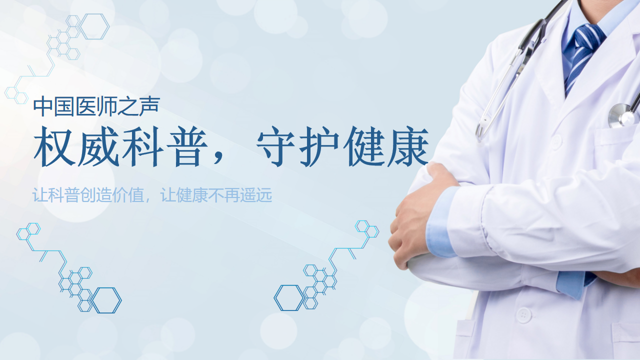
2019年1月11日，由**医学科普分会**承办的**“中国医师之声”科普平台**正式发布上线。平台科普内容以短视频为主打核心，辅以音频及图文，利用新媒体端的互动优势，**众多权威医生聚焦患者日常最关心的健康问题，深入浅出的解答医学科普知识，如“如何确定你有高血压”、“如何挑选感冒药”等。**
 医学科普分会由北京朝阳医院急诊科主任郭树彬教授牵头组建。**郭树彬教授从事急诊医学临床工作多年，对各种急危重症的诊断及治疗有着丰富的经验。在从事大量的临床工作的同时，他还独立或参加了多项高水平的科研项目，尤其在脓毒症病理生理、机体感染和免疫、代谢方面有较为深入的基础研究成果，发表第一作者文章十几篇、专业著作九部。**他认为，“很多患者的疾病原本可以通过早期治疗来达到很好的效果，因此我们希望能把健康科普知识传播给大众，让患者可以积极、正确地认识自身疾病。”
 “中国医师之声”为健康医学知识的传播提供了一个极好的平台。它秉承“大专家，小科普”的理念，汇集了上海市同济医院、北京协和医院等众多三甲医院的顶级医师为百姓奉献最权威的医学科普知识。**通过科普途径，更多医术高超、富有知识的医生与患者建立起了信息交流的渠道，对公众的疾病预防和患者疾病的早期治疗也大有裨益。**

[Expert Group]-English version

On January 11, 2019, the **"Voice of Chinese Physicians" science popularization platform**, hosted by **the Medical Science Popularization Branch**, was officially launched online. The platform's popular science content is mainly short videos, supplemented by audio and graphics. It leverages the interactive advantages of the new media platform, and **many authoritative doctors focus on the health issues that patients are most concerned about in daily life, and explain medical science popularization knowledge in a simple way, such as "how to determine if you have high blood pressure", "how to choose a cold medicine", etc.**


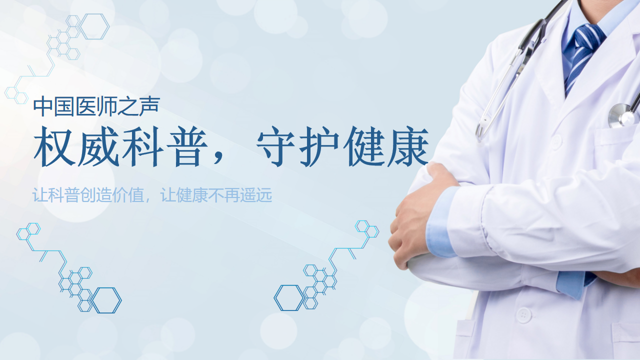
The Medical Science Popularization Branch was founded by Professor Guo Shubin, the head of the Emergency Department of Beijing Chaoyang Hospital. **Professor Guo Shubin has been engaged in clinical work in emergency medicine for many years and has rich experience in the diagnosis and treatment of various critical illnesses. While engaged in a large amount of clinical work, he has independently or participated in many high-level scientific research projects, especially in sepsis pathophysiology, body infection and immunity, metabolism, and he has relatively deep basic research results, published more than ten articles as the first author, and nine professional works.** He believes, "Many patients' diseases can achieve very good results through early treatment, so we hope to spread health science knowledge to the public so that patients can actively and correctly understand their diseases."

"Voice of Chinese Physicians" provides an excellent platform for the dissemination of health and medical knowledge. It adheres to the philosophy of "big experts, small science popularization", and gathers top doctors from many Class III A hospitals such as Tongji Hospital in Shanghai and Peking Union Medical College Hospital to contribute the most authoritative medical science knowledge to the public. **Through science popularization, more highly skilled and knowledgeable doctors have established channels for information exchange with patients, which is of great benefit to the prevention of public diseases and the early treatment of patient diseases.**

**[Angel Group]**-Chinese version

2018年8月19日，首个“中国医师节”到来，它是我国设立的第四个行业性节日。当天，来自全国的80名医师获得了国务院审批授予的第十一届“中国医师奖”，以表彰他们“**敬佑生命、救死扶伤、甘于奉献、大爱无疆**”的职业精神。获奖医师中**既有享誉国内外的名医大家和引领学科发展的院士，也有长期耕**


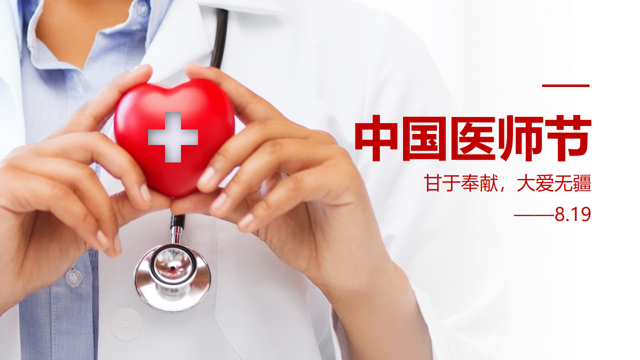
**耘在临床医疗一线的专家和扎根基层的全科及乡村医生。**
 妇产医生黄醒华就是其中一名获奖医师。从医50多年来，她亲历了新中国妇幼卫生事业的发展，亲力亲为的抢救、手术和接产上万人次，用温暖的爱心托起了万千生命。**每一位孕妇检查后起身时，她都要用手扶一下；她曾口对口吸出患有肺结核产妇嘴里的污血，保证了病人呼吸道的畅通，赢得了抢救时间；当产妇生命垂危来不及进手术室时，她跪地两个小时手术确保母婴平安；她使怀孕6次失败的农家妇女终于做了母亲；“O”型血的她多次紧急献血给被抢救的新生儿，迄今为止，八十多个孩子的身体中流动着她的热血。**她被誉为母婴生命的“守护神”、永不熄火的“救护车”。
 中国医师协会会长张雁灵表示，中国现有**注册医师360.7万人，乡村医生138万人，他们一同长年如一日践行着全心全意护佑人民健康的初心和使命。**设立“中国医师节”就是对他们坚守初心和履行使命的高度肯定。

[Angel Group]-English version

On August 19, 2018, the first "Chinese Physician's Day" arrived, which is the fourth professional holiday established in our country. On that day, 80 doctors from all over the country were awarded the 11th "Chinese Physician Award" approved by the State Council to commend their professional spirit of **"respecting life, saving the wounded, willing to dedicate, and boundless love"**. Among the award-winning doctors are **both renowned domestic and foreign medical experts and academicians leading the development of disciplines, as well as experts who have long been working on the front line of clinical medicine and general and rural doctors rooted in the grassroots.**

Obstetrician and gynecologist Huang Xinghua is one of the award-winning doctors. Over the past 50 years in medicine, she has personally experienced the development of maternal and child health in New China, and has personally saved, performed surgeries, and delivered tens of thousands of people, holding up thousands of lives with warm love. **After each pregnant woman checks up and gets up, she has to help with her hand; she has sucked out the dirty blood from the mouth of a pregnant woman with tuberculosis mouth-to-mouth, ensuring the patient's airway is unblocked, and winning rescue time; when the pregnant woman's life is in danger and she can't enter the operating room in time, she kneeled for two hours of surgery to ensure the safety of the mother and baby; she made the farmer woman who failed to get pregnant six times finally become a mother; She, with "O" type blood, has donated blood to rescued newborns in emergencies many times.** Up to now, her warm blood is flowing in the bodies of more than eighty children.


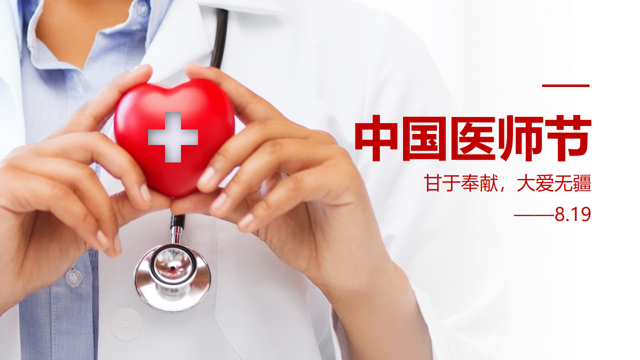
The President of the Chinese Medical Association, Zhang Yanling, stated that **there are currently 3.607 million registered doctors and 1.38 million rural doctors in China. These medical professionals have been unswervingly committed to their fundamental duty and mission of safeguarding public health, day in and day out.** The establishment of the "Chinese Doctors' Day" serves as an emphatic affirmation of their unwavering dedication and fulfillment of this noble mission.

**[Warrior Group]**-Chinese version

据2020年1月8日的新闻报道，**自除夕夜第一批医疗队到达武汉至今，全国已有346支医疗队抵达武汉和湖北，总计驰援医务人员达到4.26万人，其中重症专业的医务人员1.9万人，**此外还包括呼吸、感染、心内、肾内、心理等方面的医务人员。


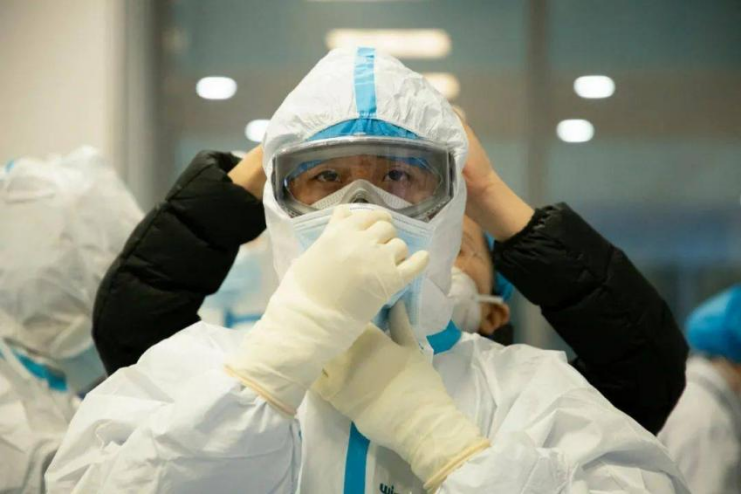
 **王婷，就是这些赶赴抗疫前线的“白衣战士”中的一员。**王婷的父亲王卫国是长医附属和平医院放射科的一名医生，2003年，王婷 70多岁的奶奶坐着轮椅把身为医生的儿子王卫国送上支援太原抗击非典的战场； 17年后的今天，已经 66岁的王卫国也同样把作为医生的女儿送上驰援湖北的抗疫一线。
 **“疫情发生后，我爸就和我说如果有机会、有可能的话一定要去一线为国家战斗。”**王婷临走前，王卫国来到现场为女儿送行，他深情地拥抱女儿：“这个时候不要掉泪，不要牵挂，家里没事，放心的去吧，安心工作。”

感人的故事还有很多，李艳艳、邢静护士因为长期戴口罩，鼻梁、耳背的皮肤都被压烂了，反复的洗手、消毒使他们的双手都开始皴裂，因为穿防护服如厕不便，她们甚至都不敢多喝水;感染科的党大夫和他的护士妻子因为双双奔赴前线，只能将两个孩子留给年迈的父母照看;每天下夜班的医护人员，脸上都留下了被口罩和护目镜长期挤压留下深深的压痕……
 **疫情无情人有情，正是有无数医护人员坚守在抗疫防疫第一线，人民群众的生命安全才多了一份保障。**

[Warrior Group]-English version

As per news reports from January 8th, 2020, **since the first medical team arrived in Wuhan on the eve of Chinese New Year, 346 medical teams from across the country have been dispatched to Wuhan and Hubei province, totaling 42,600 medical personnel, including 19,000 specialists in severe cases.** These teams encompass a variety of medical professionals specializing in fields such as respirology, infection, cardiology, nephrology, and psychology.

**
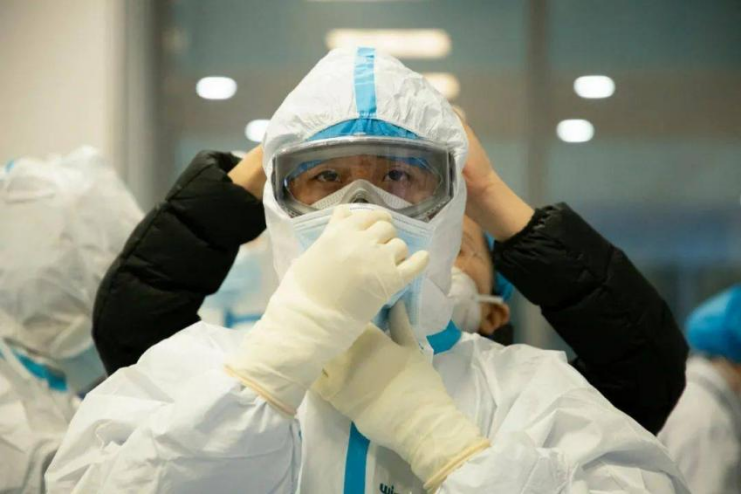
Among these white-coated warriors at the epidemic frontline is Wang Ting.** Her father, Wang Weiguo, is a radiologist at the Affiliated Peace Hospital of Chang Medical. In 2003, when Wang Ting's grandmother, then over 70 years old, sent her son, a doctor, to aid Taiyuan in combating the SARS outbreak, she was confined to a wheelchair. Now, 17 years later, Wang Weiguo, at 66 years old, has sent his own daughter, a doctor herself, to aid in the battle against the epidemic in Hubei.

**"After the epidemic started, my father told me that if there's a chance, I must go to the frontline to fight for our country,"** recalls Wang Ting. On the day of her departure, Wang Weiguo was there to see her off, hugging her warmly, "Don't shed tears at this moment, don't worry about us. Everything will be alright at home. Just go, and work with a clear mind."

There are countless moving stories of sacrifices made by medical personnel in these trying times. Nurses Li Yanyan and Xing Jing, due to prolonged usage of masks, have developed sores on their noses and behind their ears. Frequent handwashing and disinfection have led to chapped hands. As protective suits make bathroom visits inconvenient, they have reduced their water intake. Infectious disease specialist Dr. Dang and his nurse wife had to leave their children in the care of elderly parents to be on the frontline. Every night-shift medical worker bears the indelible imprint of masks and goggles on their faces, evidence of their relentless struggle against the epidemic.

**The epidemic may be merciless, but the human spirit remains resilient.** Thanks to countless medical personnel standing fast on the frontline of this battle against the epidemic, an additional layer of security is extended to the safety and lives of the people.

**[Vulnerable Group]**-Chinese version


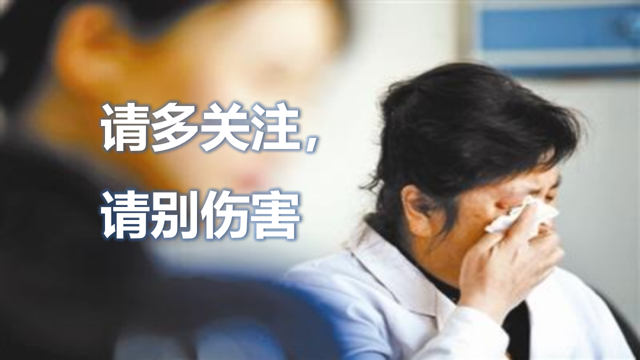
《The Lancet》公布了一项研究结果，表明国内一些年轻医生正在因为各种原因离开医院，而**医患关系紧张是影响医生离职的最主要原因。**
 **调查显示，只有 5.9%的医护人员没有遇到过医患纠纷。亲身经历过医患纠纷的医生高达 39.3%，另有54.8% 的医生曾目击同事遭遇医患纠纷。**
 一名三甲医院的医生表示：“在我工作的急诊科，一半医生被病人打过，包括我。有次后半夜值班，一位40多岁的求医者说自己有胃癌，要求开‘杜冷丁’。但他什么资料都没有，不符合开具管制药品的条件。我刚说了3个字‘不能开’，还没来得及解释原因，他就抓起桌上的瓶子砸我，还扑过来抓我。”

“听不懂医生说什么”、“住院床位紧张”等任何理由都有可能导致病人或其家属对医生**挥拳就打、拿刀就刺。**而医护人员被打、被刺后，又常常不了了之。医患纠纷的发生率居高不下，令医生们倍感紧张。
 **精神紧绷之余，医生们还必须面临高强度的工作。43.5%的医生表示每月最长留宿医院8次以上。**超负荷的工作和不规律的生活使他们多患有过劳性疾病，如肠胃炎、偏头痛等。
 **工资水平低，生活压力大也是医生们面临的一大难题。调查显示，71.6%的医生工资水平低于8000元/月，工资在10000元/月以上的医生占比仅13.1%，**许多医生兢兢业业工作多年，收入还跟不上物价涨幅，养家糊口的压力巨大。
 **“医生们的生存状态比人们想的要更加糟糕，”研究者总结道，“他们同样需要被关怀和帮助。”**

[Vulnerable Group]-English version

《The Lancet》has published a study showing that some young doctors in China are leaving hospitals for various reasons, **with tense doctor-patient relationships being the main cause of their resignations.**

**The survey revealed that only 5.9% of medical staff have never encountered a doctor-patient dispute. A staggering 39.3% of doctors have personally experienced disputes, and 54.8% have witnessed their colleagues dealing with such conflicts.**


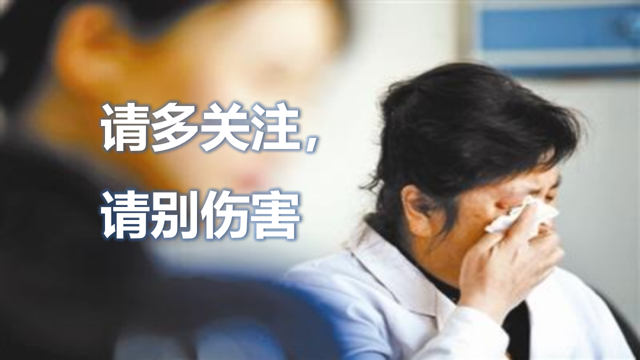
A doctor from a top-tier hospital said, "In the emergency department where I work, half of the doctors, including myself, have been hit by patients. Once during a night shift, a man in his 40s claimed he had stomach cancer and demanded a prescription for 'Duludin'. He didn't have any documentation, which was required for prescribing controlled drugs. I had barely said three words—'I can't prescribe'—before I could explain the reason, he grabbed a bottle from the table and smashed it at me and tried to grab me."

Any reason, such as "not understanding what the doctor is saying" or "a shortage of hospital beds," can lead to patients or their families hitting or even stabbing doctors. After such incidents, medical staff are often left without recourse. The high occurrence of doctor-patient disputes leaves doctors feeling increasingly tense.

**In addition to the mental strain, doctors also face high-intensity work. 43.5% of the doctors said they used to put up for the night at the hospitals more than 8 times per month.** Overloaded work and irregular lifestyles lead to them suffering from fatigue-related diseases, such as gastroenteritis and migraine.

**Low salaries and high living pressures are also major challenges for doctors.** The survey showed that 71.6% of doctors earn less than 8,000 RMB per month, and only 13.1% earn more than 10,000 RMB per month. Many doctors have worked diligently for years, but their incomes still don't keep up with the rising cost of living, resulting in enormous pressure to support their families.

**"Doctors’ living conditions are even worse than people think," the researchers concluded. "They also need care and help."**

**[Control group]**-Chinese version

8月15号下午6点多，沈阳的杨先生骑着共享单车和女朋友出去吃饭，上车时随手把自己和女朋友的包都放进了车筐里。到饭店后，杨先生拎起一个包就离开了，没注意到车筐里还有一个包。
 吃完饭，杨先生才发现自己的包没有了。包里钱不多，可证件不少。他第一时间联系了共享单车的客服人员，并通过客服查找并联系到了在杨先生之后的骑行人，但对方表示并没有看到车筐里有背包。


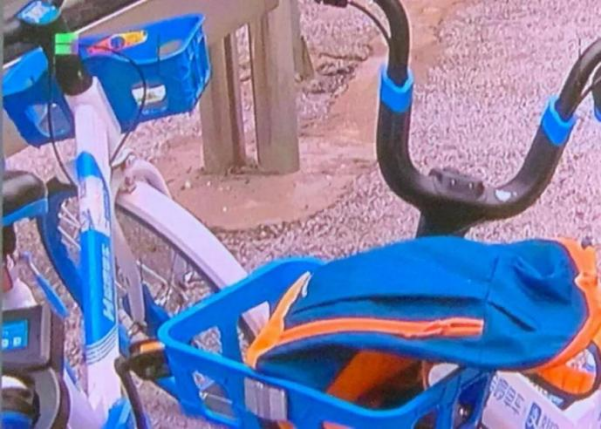


随后，杨先生拨打了110，当民警来现场调查时，饭店旁边超市的老板得知杨先生在找丢失的包，表示曾有一个小伙子，捡到一个包送到了超市里。
 “那天刚下完雨，就有个20出头的小伙进来，说在自行车上发现一个包，不知道谁落的，就把包留在我店里了，希望回来找包的人能够看到。正好这会失主过来了，确定是他的包。”
 背包失而复得，杨先生特别高兴，也非常感谢那位没有留下姓名和联系方式的好心人。杨先生还说，以后自己要是遇到类似的事情，也会站出来去帮助更多需要帮助的人。

[Control group]-English version

On the evening of October 10th, at around 9 PM, Mr. Yang, a citizen, reported to the Sungang Police Station that he had lost his backpack while dining out and needed police assistance.

Upon receiving the report, the police quickly went to the scene. Mr. Yang informed the police that at around 5 PM, he rode a shared bicycle with his girlfriend to go out for dinner. When they got on the bike, he casually placed both his and his girlfriend's bags in the bike's basket. When they arrived at the restaurant, he picked up one bag and left, not noticing that there was another bag in the basket. After finishing their meal, he realized that his bag was gone. There wasn't much money in the bag, but there were three bank cards and his ID card, and the process of reissuing the documents was very complicated, so he hoped the police could help find the bag.


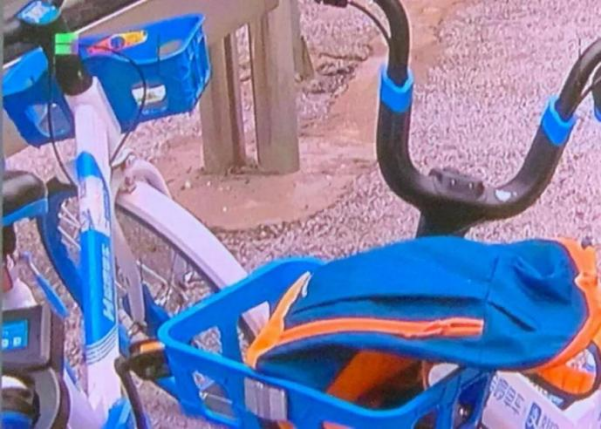
The police immediately contacted the customer service staff of the shared bicycle company and, through them, found and contacted the rider who used the bike after Mr. Yang. However, the rider said that they did not see a backpack in the basket.

Subsequently, the police conducted a visit along the route and got a clue during their investigation. The owner of a supermarket next to the restaurant learned that Mr. Yang was looking for his lost bag and said that a young man had picked up a bag and brought it to the supermarket. "It had just stopped raining that day when a young man in his early 20s came in, saying that he found a bag on a bike and didn't know whose it was. He asked if I knew. I said I didn't know, so he took the bag and left."

At present, the police have obtained the supermarket's surveillance footage and are preparing to continue the search based on the video clues. Mr. Yang is still waiting for news. The police hereby remind the public to properly secure their valuables when going out and not to place them casually. When leaving, check your belongings to avoid losing personal items.

### Appendix 2 Study 1 Questionnaire

##### [Chinese version]

**1、请您用两个词简要形容一下该新闻描述的人物形象**

**2、请根据你自己的想法在以下维度评分，1=完全不认同，5=非常认同。在你看来，医生群体的成员是？**

（1）待人热情的 1 2 3 4 5

（2）友好亲和的 1 2 3 4 5

（3）有能力的 1 2 3 4 5

（4）有才华的 1 2 3 4 5

（5）值得信赖的 1 2 3 4 5

（6）诚实正直的 1 2 3 4 5

**3、当你想到医生群体时，你会感到以下情感反应有多强烈？1=完全不，5=非常**

轻蔑 1 2 3 4 5

敬佩 1 2 3 4 5

同情 1 2 3 4 5

嫉妒 1 2 3 4 5

**4、请你根据自己想法对以下说法进行评分，1=非常不同意，5=非常同意**

1. 我相信医生不会有欺骗行为 1 2 3 4 5
2. 我对医生的工作态度非常信赖 1 2 3 4 5
3. 我信赖医疗人员的治疗技术 1 2 3 4 5
4. 我相信医生会把患者利益放在第一位 1 2 3 4 5
5. 总的来说，我信任医生 1 2 3 4 5
6. 我可以毫不犹豫地将我的生命安全交给医生 1 2 3 4 5

**5、您是否愿意做以下事情，1=非常不愿意，5=非常愿意**

1. 与医生结婚 1 2 3 4 5
2. 让你的孩子以后学医 1 2 3 4 5

**6、您的性别：**男 女

**7、您的年龄：**

**8、您的学历：**小学及以下 初中 高中 中专 大专 本科 硕士 博士

**9、您的家庭年收入：**1万元以下 1-5万 5-10万 10-20万 20万以上

**10、您目前身体状况：**1=很差 5=很好

##### [English version]

**1. Please use two words to briefly describe the character depicted in the news.**

1. **Please rate on the following dimensions according to your own thoughts, 1=strongly disagree, 5=strongly agree. In your opinion, the members of the medical community are:**

(1) Warm-hearted 1 2 3 4 5

(2) Friendly and affable 1 2 3 4 5

(3) Capable 1 2 3 4 5

(4) Talented 1 2 3 4 5

(5) Trustworthy 1 2 3 4 5

(6) Honest and upright 1 2 3 4 5

**3. How strong are your emotional responses when you think of the medical community? 1=not at all, 5=very**

(1) Contempt 1 2 3 4 5

(2) Admiration 1 2 3 4 5

(3) Sympathy 1 2 3 4 5

(4) Jealousy 1 2 3 4 5

**4. Please rate the following statements according to your own thoughts, 1=strongly disagree, 5=strongly agree**

(1) I believe that doctors will not engage in deceitful behavior 1 2 3 4 5

(2) I have great faith in the work ethic of doctors 1 2 3 4 5

(3) I trust the therapeutic skills of medical personnel 1 2 3 4 5

(4) I believe doctors will prioritize patient interests 1 2 3 4 5

(5) Overall, I trust doctors 1 2 3 4 5

(6) I can unhesitatingly entrust my life to a doctor 1 2 3 4 5

**5. Would you be willing to do the following, 1=very unwilling, 5=very willing**

(1) Marry a doctor 1 2 3 4 5

(2) Encourage your child to study medicine in the future 1 2 3 4 5

**6. Your gender:** Male; Female.

**7. Your age:**

**8. Your education:** Elementary school or below; Junior high school; High school; Vocational school; College Bachelor's degree; Master's degree; Doctorate.

**9. Your family annual income:** Below 10,000; 10,000-50,000; 50,000-100,000; 100,000-200,000; Above 200,000.

**10. Your current health condition,1=Very bad, 5=Very good:** 1 2 3 4 5

### Appendix 3: Pearson Correlation Tables for Study 1 and Study 2

##### Table S1. Pearson Correlation Tables for Study 1

|  | M | SD | 1 | 2 | 3 | 4 | 5 | 6 | 7 | 8 | 9 | 10 | 11 | 12 | 13 | 14 |
| --- | --- | --- | --- | --- | --- | --- | --- | --- | --- | --- | --- | --- | --- | --- | --- | --- |
| 1 Age | 34.670 | 7.047 | -- |  |  |  |  |  |  |  |  |  |  |  |  |  |
| 2 Gender | 0.593 | 0.492 | -0.180** | -- |  |  |  |  |  |  |  |  |  |  |  |  |
| 3 Education | 4.931 | 0.783 | -0.315*** | 0.059 | -- |  |  |  |  |  |  |  |  |  |  |  |
| 4 Income | 4.046 | 0.794 | -0.131† | 0.060 | 0.401*** | -- |  |  |  |  |  |  |  |  |  |  |
| 5 Health | 3.870 | 0.732 | -0.084 | -0.036 | 0.040 | 0.011 | -- |  |  |  |  |  |  |  |  |  |
| 6 Warmth | 3.935 | 0.719 | -0.127† | 0.024 | -0.058 | -0.080 | 0.209** | -- |  |  |  |  |  |  |  |  |
| 7 Competence | 4.310 | 0.605 | -0.184** | 0.051 | 0.105 | 0.067 | 0.11 | 0.292*** | -- |  |  |  |  |  |  |  |
| 8 Morality | 4.185 | 0.605 | -0.121† | 0.012 | 0.076 | 0.064 | 0.219** | 0.584*** | 0.386*** | -- |  |  |  |  |  |  |
| 9 Contempt | 1.250 | 0.573 | 0.127† | -0.049 | -0.158* | -0.179** | -0.031 | -0.079 | -0.258*** | -0.242*** | -- |  |  |  |  |  |
| 10 Admiration | 4.340 | 0.847 | -0.196** | 0.075 | 0.218** | 0.115† | 0.111 | 0.391*** | 0.448*** | 0.449*** | -0.232** | -- |  |  |  |  |
| 11 Sympathy | 2.970 | 1.045 | 0.004 | -0.152* | 0.060 | 0.063 | 0.006 | 0.192** | 0.056 | 0.245*** | 0.037 | 0.212** | -- |  |  |  |
| 12 Envy | 1.490 | 0.818 | 0.043 | -0.056 | -0.084 | -0.014 | 0.087 | -0.001 | -0.173* | -0.109 | 0.194** | -0.113† | 0.079 | -- |  |  |
| 13 Trust | 3.801 | 0.675 | -0.154* | -0.005 | 0.145* | 0.059 | 0.175* | 0.401*** | 0.367*** | 0.567*** | -0.276*** | 0.462*** | 0.194** | -0.124† | -- |  |
| 14 Marry | 3.560 | 0.887 | -0.045 | 0.009 | 0.049 | 0.062 | -0.014 | 0.162* | 0.167* | 0.115† | -0.064 | 0.04 | -0.101 | -0.057 | 0.220** | -- |
| 15 Children | 3.760 | 1.000 | 0.028 | 0.134* | -0.021 | 0.067 | 0.122† | 0.127† | 0.11 | 0.13† | 0.022 | 0.084 | -0.052 | 0.006 | 0.185** | 0.421*** |
| Note. †p < .10, p < .05, p < .01, p < .001.Marry = willingness to marry a doctor; Children = Encourage your child to study medicine in the future | | | | | | | | | | | | | | | | |

##### Table S2a. Correlation Matrix – Expert Portrayal Variables for Study 2

|  | α | M | SD | 1 | 2 | 3 | 4 | 5 | 6 | 7 | 8 | 9 | 10 | 11 | 12 | 13 | 14 |
| --- | --- | --- | --- | --- | --- | --- | --- | --- | --- | --- | --- | --- | --- | --- | --- | --- | --- |
| 1 Sex | -- | -- | -- | -- |  |  |  |  |  |  |  |  |  |  |  |  |  |
| 2 Age | -- | 36.160 | 13.620 | -0.290*** | -- |  |  |  |  |  |  |  |  |  |  |  |  |
| 3 Education | -- | 4.920 | 0.882 | 0.088 | -0.315*** | -- |  |  |  |  |  |  |  |  |  |  |  |
| 4 Income | -- | 3.080 | 1.121 | 0.017 | 0.263*** | 0.197*** | -- |  |  |  |  |  |  |  |  |  |  |
| 5 health | -- | 4.000 | 0.728 | 0.039 | -0.150** | 0.141* | 0.089 | -- |  |  |  |  |  |  |  |  |  |
| 6 E-Warmth | 0.721 | 3.883 | 0.632 | -0.013 | -0.023 | -0.017 | -0.006 | 0.156** | -- |  |  |  |  |  |  |  |  |
| 7 E-Competence | 0.733 | 4.480 | 0.534 | 0.066 | -0.083 | 0.016 | 0.050 | 0.093† | 0.415*** | -- |  |  |  |  |  |  |  |
| 8 E-Morality | 0.719 | 4.261 | 0.572 | -0.019 | -0.063 | 0.086 | 0.056 | 0.115* | 0.542*** | 0.476*** | -- |  |  |  |  |  |  |
| 9 E-Contempt | -- | 1.220 | 0.533 | -0.103† | 0.131* | -0.049 | -0.010 | -0.127* | -0.152** | -0.193** | -0.218*** | -- |  |  |  |  |  |
| 10 E-Adimiration | -- | 4.310 | 0.748 | 0.053 | -0.044 | 0.124* | 0.036 | 0.111* | 0.352*** | 0.459*** | 0.452*** | -0.210*** | -- |  |  |  |  |
| 11 E-Sympathy | -- | 2.630 | 0.994 | -0.067 | 0.025 | -0.031 | -0.039 | 0.154** | 0.115* | 0.024 | 0.035 | 0.096† | 0.093† | -- |  |  |  |
| 12 E-Envy | -- | 1.330 | 0.640 | -0.129* | 0.024 | -0.036 | -0.135* | -0.065 | -0.188** | -0.187** | -0.230*** | 0.423*** | -0.101† | 0.056 | -- |  |  |
| 13 E-Trust | 0.846 | 4.124 | 0.534 | 0.02 | -0.005 | 0.057 | 0.067 | 0.187** | 0.528*** | 0.485*** | 0.674*** | -0.242*** | 0.549*** | 0.078 | -0.248*** | -- |  |
| 14 E-Marry | -- | 3.620 | 0.994 | -0.022 | 0.173** | -0.007 | 0.111* | 0.098† | 0.121* | 0.133* | 0.230*** | 0.004 | 0.138* | -0.050 | -0.054 | 0.302*** | -- |
| 15 E-Children | -- | 3.740 | 0.995 | -0.067 | 0.124* | 0.054 | 0.065 | 0.111* | 0.205*** | 0.114* | 0.189** | -0.004 | 0.194*** | -0.024 | 0.042 | 0.289*** | 0.570*** |
| Note. †p < .10, p < .05, p < .01, p < .001. E = score for Expert doctor portrayal; A = score for Angel doctor portrayal; V = score for Vulnerable doctor portrayal.Marry = willingness to marry a doctor; Children = Encourage your child to study medicine in the future | | | | | | | | | | | | | | | | | |

##### Table S2b. Correlation Matrix – Angel Portrayal Variables for Study 2

|  | α | M | SD | 1 | 2 | 3 | 4 | 5 | 16 | 17 | 18 | 19 | 20 | 21 | 22 | 23 | 24 |
| --- | --- | --- | --- | --- | --- | --- | --- | --- | --- | --- | --- | --- | --- | --- | --- | --- | --- |
| 1 Sex | -- | -- | -- | -- |  |  |  |  |  |  |  |  |  |  |  |  |  |
| 2 Age | -- | 36.160 | 13.620 | -0.290*** | -- |  |  |  |  |  |  |  |  |  |  |  |  |
| 3 Education | -- | 4.920 | 0.882 | 0.088 | -0.315*** | -- |  |  |  |  |  |  |  |  |  |  |  |
| 4 Income | -- | 3.080 | 1.121 | 0.017 | 0.263*** | 0.197*** | -- |  |  |  |  |  |  |  |  |  |  |
| 5 health | -- | 4.000 | 0.728 | 0.039 | -0.150** | 0.141* | 0.089 | -- |  |  |  |  |  |  |  |  |  |
| 16 A-Warmth | 0.802 | 4.145 | 0.707 | -0.038 | -0.064 | -0.021 | 0.042 | 0.120* | -- |  |  |  |  |  |  |  |  |
| 17 A-Competence | 0.636 | 4.338 | 0.530 | 0.045 | -0.089 | 0.039 | 0.034 | 0.190** | 0.423*** | -- |  |  |  |  |  |  |  |
| 18 A-Morality | 0.700 | 4.327 | 0.592 | -0.019 | -0.089 | 0.042 | 0.029 | 0.199*** | 0.666*** | 0.487*** | -- |  |  |  |  |  |  |
| 19 A-Contempt | -- | 1.230 | 0.591 | -0.101† | 0.103† | -0.011 | 0.013 | -0.027 | -0.176** | -0.198*** | -0.206*** | -- |  |  |  |  |  |
| 20 A-Adimiration | -- | 4.420 | 0.725 | 0.042 | -0.125* | 0.093† | 0.010 | 0.115* | 0.443*** | 0.341*** | 0.528*** | -0.295*** | -- |  |  |  |  |
| 21 A-Sympathy | -- | 2.850 | 1.029 | -0.110† | 0.053 | 0.028 | 0.071 | 0.079 | 0.106† | 0.017 | 0.082 | 0.090 | 0.141* | -- |  |  |  |
| 22 A-Envy | -- | 1.270 | 0.587 | -0.050 | 0.107† | -0.048 | -0.054 | -0.064 | -0.146** | -0.163** | -0.165** | 0.302*** | -0.220*** | 0.096† | -- |  |  |
| 23 A-Trust | 0.840 | 4.177 | 0.524 | 0.048 | 0.018 | -0.004 | 0.095 | 0.167** | 0.579*** | 0.470*** | 0.673*** | -0.271*** | 0.631*** | 0.094† | -0.123* | -- |  |
| 24 A-Marry | -- | 3.610 | 1.036 | -0.081 | 0.163** | 0.027 | 0.099† | 0.082 | 0.076 | 0.109† | 0.121* | -0.069 | 0.178** | 0.005 | 0.096† | 0.242*** | -- |
| 25 A-Children | -- | 3.650 | 1.021 | -0.062 | 0.111* | 0.073 | 0.108† | 0.146** | 0.127* | 0.149** | 0.138* | -0.009 | 0.143* | 0.029 | 0.056 | 0.240*** | 0.580*** |
| Note. †p < .10, p < .05, p < .01, p < .001. E = score for Expert doctor portrayal; A = score for Angel doctor portrayal; V = score for Vulnerable doctor portrayal.Marry = willingness to marry a doctor; Children = Encourage your child to study medicine in the future | | | | | | | | | | | | | | | | | |

#### Table S2c. Correlation Matrix – Vulnerable Portrayal Variables for Study 2

|  | α | M | SD | 1 | 2 | 3 | 4 | 5 | 26 | 27 | 28 | 29 | 30 | 31 | 32 | 33 | 34 |
| --- | --- | --- | --- | --- | --- | --- | --- | --- | --- | --- | --- | --- | --- | --- | --- | --- | --- |
| 1 Sex | -- | -- | -- | -- |  |  |  |  |  |  |  |  |  |  |  |  |  |
| 2 Age | -- | 36.160 | 13.620 | -0.290*** | -- |  |  |  |  |  |  |  |  |  |  |  |  |
| 3 Education | -- | 4.920 | 0.882 | 0.088 | -0.315*** | -- |  |  |  |  |  |  |  |  |  |  |  |
| 4 Income | -- | 3.080 | 1.121 | 0.017 | 0.263*** | 0.197*** | -- |  |  |  |  |  |  |  |  |  |  |
| 5 health | -- | 4.000 | 0.728 | 0.039 | -0.150** | 0.141* | 0.089 | -- |  |  |  |  |  |  |  |  |  |
| 26 V-Warmth | 0.798 | 3.825 | 0.677 | -0.019 | -0.048 | 0.005 | -0.011 | 0.215*** | -- |  |  |  |  |  |  |  |  |
| 27 V-Competence | 0.721 | 4.259 | 0.567 | 0.045 | -0.160** | 0.052 | 0.072 | 0.162** | 0.388*** | -- |  |  |  |  |  |  |  |
| 28 V-Morality | 0.655 | 4.111 | 0.580 | 0.08 | -0.116* | 0.076 | 0.082 | 0.186** | 0.608*** | 0.515*** | -- |  |  |  |  |  |  |
| 29 V-Contempt | -- | 1.240 | 0.543 | -0.105† | 0.159** | -0.051 | 0.039 | -0.014 | -0.142* | -0.165** | -0.278*** | -- |  |  |  |  |  |
| 30 V-Adimiration | -- | 4.200 | 0.754 | 0.105† | -0.191** | 0.180** | -0.020 | 0.121* | 0.401*** | 0.407*** | 0.508*** | -0.231*** | -- |  |  |  |  |
| 31 V-Sympathy | -- | 3.420 | 1.047 | -0.037 | -0.010 | 0.095† | 0.005 | 0.014 | 0.188** | -0.03 | 0.140* | 0.012 | 0.326*** | -- |  |  |  |
| 32 V-Envy | -- | 1.310 | 0.600 | -0.171** | 0.086 | -0.124* | -0.049 | -0.048 | -0.212*** | -0.156** | -0.280*** | 0.465*** | -0.277*** | -0.064 | -- |  |  |
| 33 V-Trust | 0.830 | 4.064 | 0.531 | 0.035 | -0.045 | 0.108† | 0.063 | 0.211*** | 0.574*** | 0.457*** | 0.660*** | -0.208*** | 0.536*** | 0.248*** | -0.212*** | -- |  |
| 34 V-Marry | -- | 3.470 | 1.068 | -0.056 | 0.116* | 0.044 | 0.103† | 0.074 | 0.152** | 0.152** | 0.200*** | -0.024 | 0.149** | -0.068 | 0.061 | 0.248*** | -- |
| 35 V-Children | -- | 3.500 | 1.117 | -0.039 | 0.077 | 0.111* | 0.117* | 0.121* | 0.238*** | 0.167** | 0.207*** | -0.009 | 0.202*** | -0.042 | -0.022 | 0.270*** | 0.628*** |
| Note. †p < .10, p < .05, p < .01, p < .001. E = score for Expert doctor portrayal; A = score for Angel doctor portrayal; V = score for Vulnerable doctor portrayal.Marry = willingness to marry a doctor; Children = Encourage your child to study medicine in the future | | | | | | | | | | | | | | | | | |
